# Low nanomolar affinity to major grass pollen allergen Phl p 5 as achieved in an unmutated human antibody-lineage ancestor

**DOI:** 10.3389/fimmu.2025.1600778

**Published:** 2025-07-01

**Authors:** Mattias Essén, Eric Franciskovic, Céleste Sele, Magdalena Godzwon, Mats Ohlin

**Affiliations:** ^1^ Department of Immunotechnology, Lund University, Lund, Sweden; ^2^ Lund Protein Production Platform LP3, Department of Biology, Lund University, Lund, Sweden; ^3^ SciLifeLab, Lund University, Lund, Sweden

**Keywords:** affinity, antibody, antibody repertoire, IgE, immunoglobulin germline gene allele, molecular evolution, somatic hypermutation, unmutated common ancestor (UCA)

## Abstract

**Background:**

Group 5 allergens, such as Phl p 5 of timothy grass, are major contributors to grass pollen allergy. Antibody 212597 specific for this allergen was recently isolated by single cell sequencing of bone marrow B cells of a grass pollen-allergic subject. This antibody, although subjected only to a low level of hypermutation resulting in six amino acid substitution across the heavy and light chain variable domains, has achieved sub-nM affinity for the allergen, suggesting that antibodies specific for this major group of allergens can be of high affinity even at the naïve, unmutated stage. The present study was designed to assess affinity and biophysical characters of the antibody, its inferred unmutated ancestor, and other intermediate and allelic variants thereof.

**Methods:**

Site-directed mutagenesis was used to revert substitutions of antibody 212579. Mutants, including its inferred unmutated common ancestor were characterized with respect to allergen affinity, thermostability, and hydrodynamic radius.

**Results:**

We demonstrate that even the antibody’s inferred unmutated common ancestor shows high affinity for the allergen in the low-nM range. Glutamate at heavy chain position 38, a residue unique to allele IGHV3-48*03, the germline gene origin of the heavy chain of antibody 212579, was critical for high affinity binding. Substitution to serine as found in other alleles of IGHV3–48 reduced the affinity about 20-fold. A substitution, N40_H_T in the heavy/light chain variable domain interface, introduced into the antibody through somatic hypermutation, did not impact its affinity for the allergen but reduced its thermal stability and increased its hydrodynamic radius.

**Conclusion:**

Unmutated, high affinity (low-nM) antibodies specific for a major allergen (Phl p 5) can be generated directly in naïve B cells and are, given an appropriate rearrangement, imprinted into the repertoire through rearrangements involving immunoglobulin germline gene alleles IGHV3-48*03 and IGKV3-20*01. This specificity depends on an allele-unique residue encoded by the immunoglobulin germline repertoire. Substitutions in the heavy/light chain variable domain interface, such as N40_H_T in a heavy chain variable domain, might negatively impact biophysical properties of the antibody and should be considered as targets for further evolution or reversion if they negatively impact an antibody’s developability properties.

## 1 Introduction

Allergen-specific antibodies are key to the induction, but also the resolution, of allergic disease (1). Studies of such antibodies and their development have been hampered by the rarity of allergen-specific B cells, in particular those producing IgE, the hallmark antibody isotype that cause the allergic condition. The origins of IgE-producing cells and the memory compartment from which IgE-producing cells are derived have also been matters of controversy (2). Looney et al. (3) demonstrated through use of large-scale next generation sequencing that IgG1-producing cells were the most likely origins of IgE-producing cells. Lately, the IgG-producing compartment was confirmed to contribute substantially to IgE memory (4–6). There is thus a strong link between the population of antibodies of the IgG and IgE isotype and the latter may develop from memory residing in a subpopulation of the memory cells that encode the allergen-specific IgG1 compartment.

High affinity antigen recognition, a feature commonly important for the potency of antibodies including those specific for allergens (7, 8), is typically achieved through processes of somatic hypermutation and selection in secondary lymphoid structures. However, it is conceivable that high affinity can be imprinted into an antibody already at the unmutated, naïve stage, suggesting that highly functional antibodies can be developed early during a humoral immune response. Our limited knowledge of allergen-specific antibody repertoires similarly limit our understanding of such processes.

**Figure 1:**
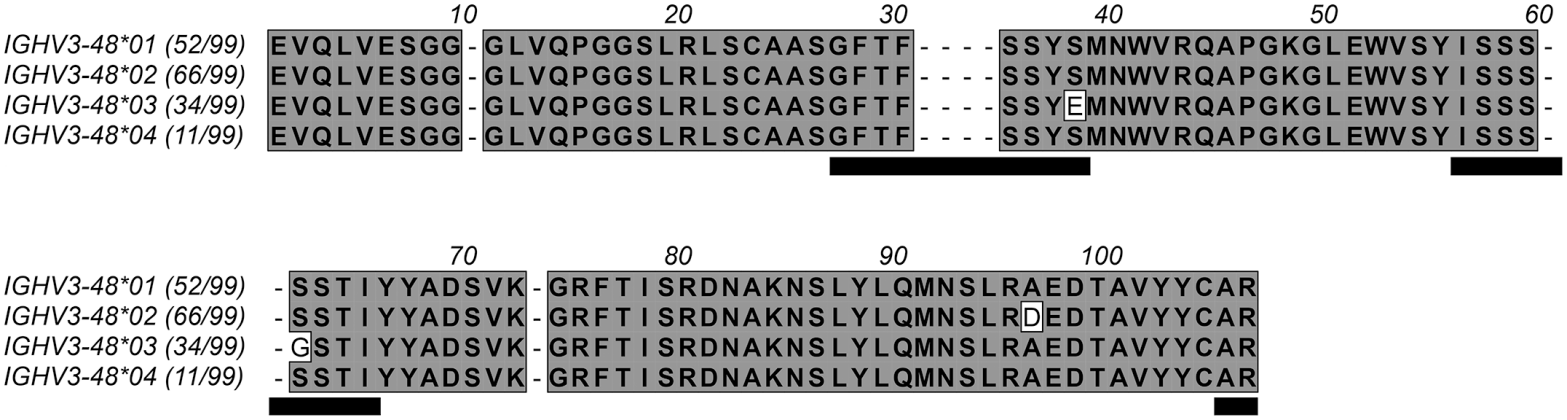
Protein sequences encoded by the four alleles of IGHV3-48. The number of subjects among 99 individuals in a Norwegian cohort (17) that encode antibodies from these alleles (https://vdjbase.org) are shown in parenthesis after each allele name. Residues are numbered according to the IMGT numbering system (13). Gaps (represented by dashes) are introduced into the sequences to account for residues that are present in some products of immunoglobulin and T cell receptor genes but absent in products derived from IGHV3–48 according to this numbering system. Residues belonging to CDRs are underlined. Residues 38_H_ and 62_H_, which both vary among products encoded by the alleles of IGHV3-48, locate to CDR1 and CDR2, respectively. The D96_H_ variant of IGHV3-48*02 is in the loop connecting the E and F β-strands, on the back side of the variable domain far from the antibody paratope.

Single cell sequencing technology, offers opportunities for in-depth studies of immunity and has been used to define native human allergen-specific antibodies (9–11). Such an approach was recently used to identify grass pollen-specific antibodies. One such high affinity (sub-nM) antibody, clone 212579, specific for grass pollen group 5 allergens, like the major timothy allergen Phl p 5 (12), was encoded by a bone marrow (BM)-derived cell (9). This B cell lineage produced antibodies of both the IgE isotype and IgG1 subclass. The fully sequenced IgG1 antibody was minimally mutated (9). In contrast, the partially sequenced set of IgE antibodies, also encoded by cells of BM, was substantially more mutated (9). The antibody clonotype was derived from the commonly utilized light chain allele IGKV3-20*01 and the heavy chain germline allele IGHV3-48*03, the products of which differ from other more commonly used alleles of the IGHV3–48 gene in two residues of the heavy chain complementary determining regions (CDR) (**Figure 1**). Altogether this clonotype offers an opportunity for detailed assessment of (1) the potential to generate high affinity group 5 pollen allergen-specific antibodies already at the unmutated, naïve stage (2), the path for development of a high affinity allergen-specific clonotype, and (3) the role of allele-differentiating diversity for the generation of an allergen-specific antibody. We demonstrate that the inferred unmutated common ancestor (UCA) of antibody 212579 maintained a high (low-nM) affinity for the allergen illustrating the ability of the naïve repertoire to generate high affinity antibody towards the major allergen Phl p 5. We also demonstrate that an immunoglobulin heavy chain variable allele-specific residue is important for establishing the sub-nM affinity of antibody 212579.

## 2 Methods

### 2.1 The 212579 Phl p 5-specific human monoclonal antibody

**Figure 2:**
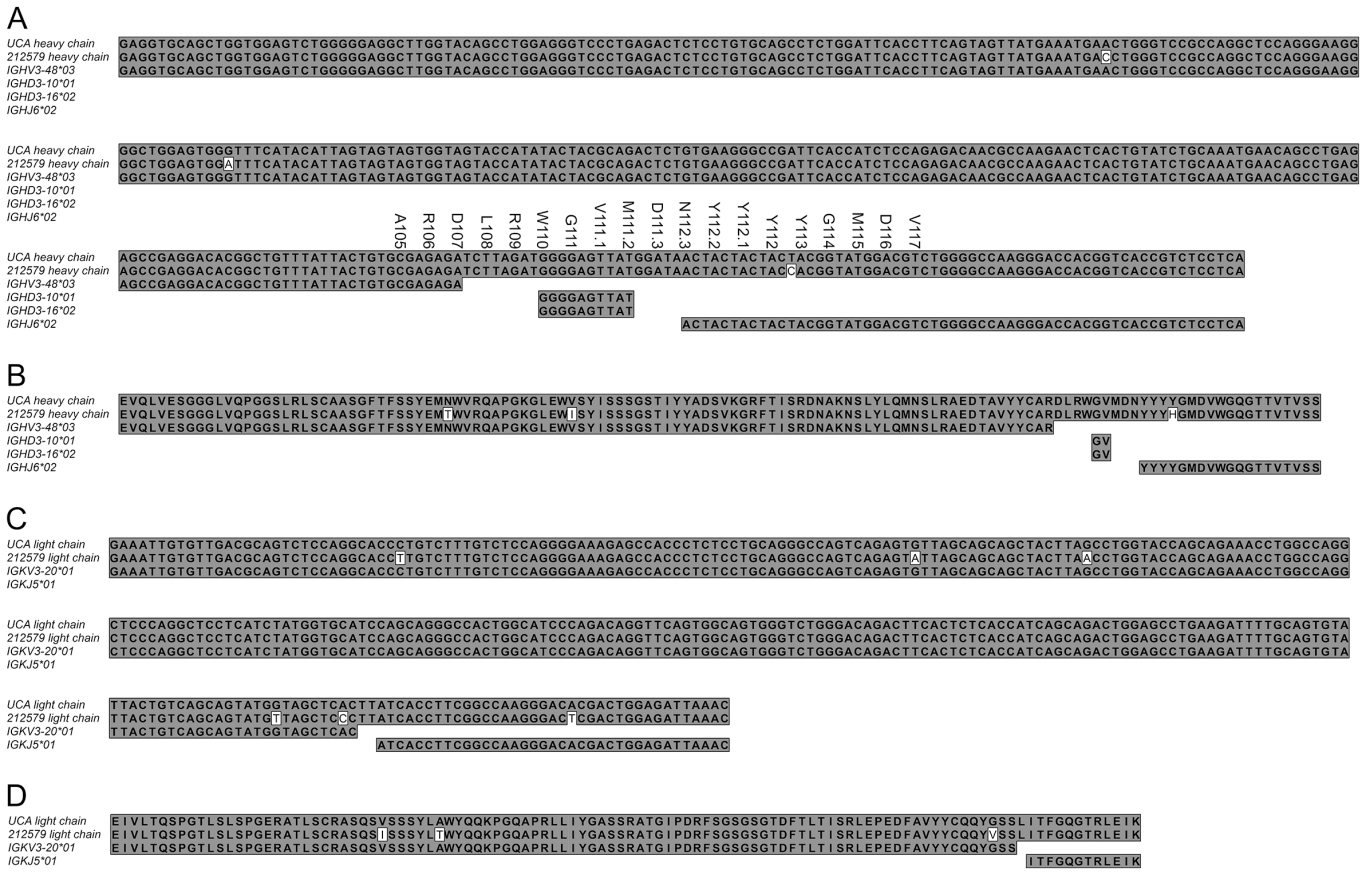
Nucleotide and protein sequences of the heavy **(A, B)** and light **(C, D)** chain variable domains of the human Phl p 5-specific antibody 212579 and its most likely inferred UCA. The sequence and residue numbers of CDR3 of the heavy chain of the inferred UCA is spelled out above the nucleotide sequence **(A)**. The parts of the variable, diversity, and joining germline genes that contributed to the rearrangements in the heavy and kappa light chain loci are shown **(A, C)**. Only amino acids that were fully encoded by the incorporated genes are spelled out in panels **(B, D)** In addition, two bases of germline genes were incorporated into codons encoding D107_H_, W110_H_, M111.2_H_, and N112.3_H_ and one base of germline IGKV3-20*1 were incorporated into the codon encoding L115_L_.

The high (sub-nM) affinity human monoclonal IgG1 antibody 212579 was previously identified by single cell sequencing of antibody encoding genes of bone marrow cells of a grass-pollen allergic subject (9). Clonally related IgE heavy chain-encoding transcripts were identified in the same subject (9). Genes encoding the heavy and light chain of antibody 212579 (**Figure 2**) are available using GenBank accession numbers PQ539330 and PQ539331. Residues are numbered according to the IMGT numbering system (13). For instance, E38_H_ refers to a glutamate residue at position 38 of the heavy (_H_) chain, and V29_L_I refers to a substitution of residue 29 of the light (_L_) chain from valine to isoleucine.

### 2.2 Mutagenesis

**Figure 3:**
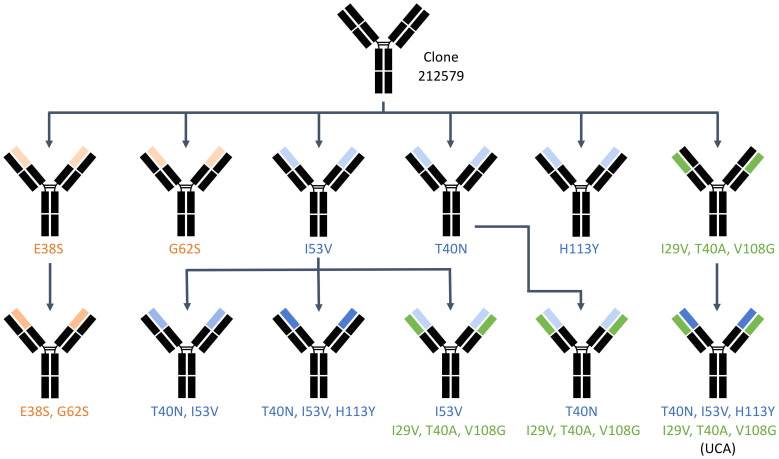
Experimental paths through which the different variants of 212579 were generated by the site-directed mutagenesis process. Heavy chain domain variants are highlighted in blue (those related to reversion of substitutions that had been introduced by mutation of the inferred UCA) and orange (those related to sequence differences between alleles of IGHV3-48). Domains carrying reversion of substitutions of the light chain are highlighted in green.

Codon optimized genes (GenBank accession numbers PQ539345 and PQ539358) encoding the heavy and light chain variable domains of antibody 212579 were previously cloned into a eukaryotic expression vector and expressed as IgG1 (9). Site-directed mutagenesis was performed using the GeneArt^®^ Site-Directed Mutagenesis System (Thermo Fisher Scientific, Waltham, MA, USA) for single mutations and the GeneArt^®^ Site-Directed Mutagenesis PLUS Kit for multi-site mutagenesis using primers listed in **Table 1**. PCR conditions included an initial denaturation at 94°C for 2 minutes, followed by 18 cycles at 94°C for 20 seconds, 57°C for 30 seconds, and 68°C for 3 minutes, with a final extension at 68°C for 5 minutes. Mutant constructs were verified via Sanger sequencing (Eurofins Genomics, Ebersberg, Germany). Clones were experimentally derived by a single mutagenesis process or in a sequential manner, as outlined in **Figure 3**.

**Table 1:** Primers used to generate variants of antibody 212579.

Substitution	Primers
T40_H_N	GCTCCTACGAGATGAACTGGGTTAGACAGGC GCCTGTCTAACCCAGTTCATCTCGTAGGAGC
I53_H_V	AAAGGCCTGGAATGGGTCAGCTACATCAGCA TGCTGATGTAGCTGACCCATTCCAGGCCTTT
H113_H_Y	GATAACTACTATTACTACGGCATGGATGTGT ACACATCCATGCCGTAGTAATAGTAGTTATC
I29_L_V, T40_L_A	AGAGCTTCTCAGAGCGTCAGCAGCAGCTACCTGGCATGGTATCAGCAAA TTTGCTGATACCATGCCAGGTAGCTGCTGCTGACGCTCTGAGAAGCTCT
V108_L_G	ACTGCCAGCAGTACGGGTCCTCCCTGATCAC GTGATCAGGGAGGACCCGTACTGCTGGCAGT
E38_H_S	ACCTTCAGCTCCTACTCGATGACCTGGGTTAG CTAACCCAGGTCATCGAGTAGGAGCTGAAGGT
G62_H_S	TACATCAGCAGCTCTAGCAGCACAATCTACT AGTAGATTGTGCTGCTAGAGCTGCTGATGTA

### 2.3 Protein production and purification

Plasmids encoding antibody variants were amplified in DH5α *Escherichia coli* (Thermo Fisher Scientific) and purified using the EndoFree Plasmid Maxi Kit (Qiagen, Hilden, Germany) following the manufacturer’s protocol. Expi293F™ suspension cells (Thermo Fisher Scientific) were cultured in Expi293™ Expression Medium (Gibco™, Carlsbad, CA, USA) under conditions optimized for exponential growth (37°C, 8% CO_2_, 225 rpm). Cells were maintained in 125 mL shake flasks at a volume of 30 mL and passaged every 3–4 days when reaching a density of 3–5 × 10^6^ cells/mL by diluting cultures to 0.3–0.5 × 10^6^ cells/mL. For transfection, cells were seeded at 2.5 × 10^6^ cells/mL in fresh Expi293™ Expression Medium one day prior. On the day of transfection, cells were adjusted to a final concentration of 3 × 10^6^ cells/mL and seeded at 2.5 mL per well in a 24-well plate, allowing cells to settle for 4 hours before transfection. Plasmid DNA (2.5 µg per well) and PEI MAX Reagent (1 mg/mL) (PEI MAX^®^ - Transfection Grade Linear Polyethylenimine Hydrochloride (MW 40,000), Polysciences, Inc., Warrington, PA, USA) were diluted in sterile-filtered (0.22 µm) 0.15 M NaCl. DNA and PEI were mixed at DNA: PEI ratios of 1:6 and 1:9 (w/w), gently swirled, and incubated at room temperature for 10–20 minutes to ensure complex formation. The DNA: PEI complexes were added dropwise to the wells containing cells while swirling to promote even distribution. Cells were incubated at 37°C, 8% CO_2_, and 225 rpm for six days before harvest of culture supernatant.

Antibodies were purified using Pierce™ Protein A Magnetic Beads (Thermo Fisher Scientific, Product No. 88845) according to the manufacturer’s protocol. Briefly, a 900 µL aliquot of clarified supernatant was combined with 100 µL of 10X binding/wash buffer (200 mM Tris, 1.5 M NaCl, 0.5% Tween-20, pH 7.4) in a 24-well deep-well plate with pointy-tip wells. Beads (50 µL per sample) were pre-washed with 150 µL of 1X binding/wash buffer and magnetically separated before incubation with the diluted supernatant at room temperature for 1 hour with gentle mixing. Following incubation, beads were washed twice with 500 µL of 1X binding/wash buffer to remove unbound proteins. Bound antibodies were eluted by adding 100 µL of 0.1 M glycine (pH 2.0) and incubating for 10 minutes with occasional mixing. The eluate was immediately neutralized with 15 µL of 1 M Tris (pH 8.5) and stored at 4°C. Antibody concentration was determined at 280 nm using a NanoDrop ND-1000 UV-Vis Spectrophotometer using an extinction coefficient (ϵ) of 1.4 (mg/mL)−¹ cm−¹.

### 2.4 Immunochemical analysis

Indirect chemiluminescent ELISA assessed antibody binding to antigen Phl p 5. A 96 well plate (Corning, Corning, NY, USA) was coated overnight at 4°C with Phl p 5.0101 (Biomay, Vienna, Austria) diluted to 0.5 μg/mL with cold PBS (Dulbecco’s Phosphate Buffered Saline without Ca^2+^ and Mg^2+^ (Cytiva, Marlborough, MA, USA)). Washing steps were performed with a solution of 150 mM NaCl with 0.05% Tween 20, and a blocking step (after coating) was done with 0.5% BSA in PBS with 0.05% Tween 20. Incubation for each step except the coating was done at room temperature for 1 hour on the shaking table. Investigated antibodies were incubated with the immobilized antigen and detected with horse radish peroxidase (HRP)-conjugated goat anti-human IgG (Invitrogen, Carlsbad, CA, USA) and SuperSignal ELISA Pico Chemiluminescent Substrate (Thermo Scientific). Luminescence was measured using a FLUOstar Omega plate reader (BMG Labtech, Ortenberg, Germany).

### 2.5 SDS-PAGE

SDS-PAGE was performed to evaluate the purity and integrity of produced IgG. Proteins were mixed with 4× LDS Sample Buffer (Clear Page, C.B.S. Scientific) and 10× Sample Reducing Agent (Novex, Life Technologies), heated at 70°C for 10 minutes, and separated on a 10% Bis-Tris gel (NuPAGE™, Life Technologies). A pre-stained protein ladder (PageRuler, Thermo Scientific) was included for molecular weight estimation. Electrophoresis was carried out in MES SDS Running Buffer (Invitrogen) at 200 V for 35 minutes. Gels were stained with SimplyBlue™ SafeStain (Invitrogen) for 1 hour and destained in deionized water overnight. Band patterns were visualized using a Gel Doc EZ Imager (BioRad, Hercules, CA, USA).

### 2.6 Determination of antigen binding affinity and reaction rate constants

Surface plasmon resonance (SPR) was used to assess the binding kinetics and affinity of antibodies for Phl p 5, using a Biacore 1K+ instrument (Cytiva, Uppsala, Sweden). Anti-human IgG (Fc) (the Human Antibody Capture Kit (Cytiva, Product No. BR100839)) were immobilized on a CM5 sensor chip (Cytiva) anti human IgG was captured on this surface. The capture level was optimized to reach approximately 100 response units (RU). Binding experiments were conducted in PBS-based running buffer containing 0.02% Tween 20. Serial dilutions of Phl p 5 antigen (0 nM, and 1.875–60 nM) in a twofold dilution series were used. Each antigen concentration was injected at 25 µL/min for 120 seconds, followed by a 1360-second dissociation phase. The sensor surface was regenerated using 3 M MgCl_2_ (Cytiva). Binding kinetics were determined using Biacore Insight software (Cytiva) and data were fitted to a 1:1 Langmuir binding model.

### 2.7 Determination of antibody thermostability

Antibody thermal stability was assessed using Nano Differential Scanning Fluorimetry (NanoDSF, Prometheus (Nanotemper, Munich, Germany)). Antibody samples were loaded into NanoDSF standard capillaries and subjected to a temperature gradient (rate: 1°/min) from 25°C to 95°C. Intrinsic tryptophan fluorescence at 330 nm and 350 nm was recorded and melting temperature (Tm) values were determined from the first derivative of the unfolding curve. Measurements were performed in triplicates.

### 2.8 Determination of antibody hydrodynamic radius

The hydrodynamic radius (R_h) and sample monodispersity were assessed using the Fida Neo system (FidaBio, Copenhagen, Denmark). Antibody samples, stored at 4°C prior to analysis, were analyzed in triplicate using a reversibly coated hydrophilic capillary. Measurements were conducted at 25°C and 37°C, with the original antibody buffer serving as both the running and wash buffer. Data analysis employed single- or dual-species fitting models to assess sample homogeneity and detect potential aggregation or structural changes at different temperatures.

### 2.9 Structure models and structures

Five structure models of antibody 212579 and its inferred UCA as single chain fragment variable (scFv), with a ((G_4_S)_3_ linker) were generated using AlphaFold3 (https://alphafoldserver.com/) (14). A single structure model of 212579 and its inferred UCA as Fv were generated using ABodyBuilder2 (15) featured on the Bionamic software platform for antibody discovery and development (https://bionamic.io/). Model structures were visualized using the PyMOL Molecular Graphics System, Version 3.0.3 (Schrödinger, LLC, Mannheim, Germany). Structure models generated by AlphaFold3 were aligned to models generated by ABodyBuilder2 taking Cα of residues not being part of heavy chain CDR3 into account.

Five crystal structures (PDB: 2UZI, 5D70, 5TY6, 6PE7, and 6PHG) of human antibodies with an origin in IGHV3–48 or related germline genes with N40_H_ and with a resolution of ≤2.5 Å were identified from the IMGT 3D-structure database (16). Structures were visualized using the PyMOL Molecular Graphics System, Version 3.0.3.

### 2.10 Immunoglobulin heavy chain variable alleles in the human population

The occurrences of alleles of IGHV3–48 were assessed in a Norwegian cohort defined by immunoglobulin transcriptome sequencing extensively investigated in the past (17–19). Data describing this cohort is available in the VDJbase online database (20).

### 2.11 General mutation patterns of antibodies derived from IGHV3-48*03

Theoretical mutation patterns of sequences derived from alleles of IGHV3–48 at the 10% mutational frequency level had been computed using the ARMADiLLO online tool (https://armadillo.dhvi.duke.edu) (21, 22). Mutational patterns of residues 40 and 53 of human IgG derived from IGHV3–48 found in bone marrow were computed using data generated by next generation sequencing of immunoglobulin-encoding transcriptomes, as described (23, 24).

## 3 Results

### 3.1 The mutational status of Phl p 5-specific human antibody 212579 and definition of the lineage’s unmutated common ancestor

The heavy chain variable domains of antibody 212579 has an origin in germline genes IGHV3-48*03, IGHD3-10*01 or IGHD3-16*02, IGHJ6*02, IGKV3-20*01, and IGKJ5*01 (9). Analysis of the sequence defined, assuming the absence of mutations among the non-templated nucleotides of the rearrangement, a most likely UCA (**Figure 2**) (GenBank accession numbers PV255645, and PV255646). Only two bases of the IGKV-IGKJ rearrangement are not covered by the germline genes. The CDR3 of the heavy chain is 19 residues long. Only three of its residues (L108_H_, R109_H_, and D111.3_H_) are not encoded by any base of the IGHV, IGHD, or IGHJ genes. Altogether, the 212579 IgG1 sequence carry only three substitutions (N40_H_T, V53_H_I, and Y113_H_H) of the heavy chain and three of the light chain (V29_L_I, A40_L_T, and G108_L_V) relative the inferred UCA demonstrating the limited extent of mutation that the genes had undergone. These findings together suggested a high confidence in the definition of the inferred UCA sequence. Sequences of transcripts encoding the region from residue 24 to the end of the heavy chain variable domain of the related IgE identified the presence of multiple additional substitutions, including A24_H_V, S35_H_N, S59_H_G, I65_H_L, K84_H_N, Y88_H_S, N92_H_K, D111.3_H_E, N112.3_H_H, and H113_H_N, demonstrating the further extensive evolution of this clonotype into the form represented by an IgE clone that co-existed with the IgG1 clone at the time of sample collection. Both the minimally mutated antibody 212579 and the IgE-variant thereof carrying additional mutations in the heavy chain corresponding to those identified in IgE-encoding transcripts showed sub-nM affinity for Phl p 5 (9).

### 3.2 The 212579 antibody, its unmutated common ancestor, and potential intermediate sequence variants and their allergen-binding characteristics

**Figure 4:**
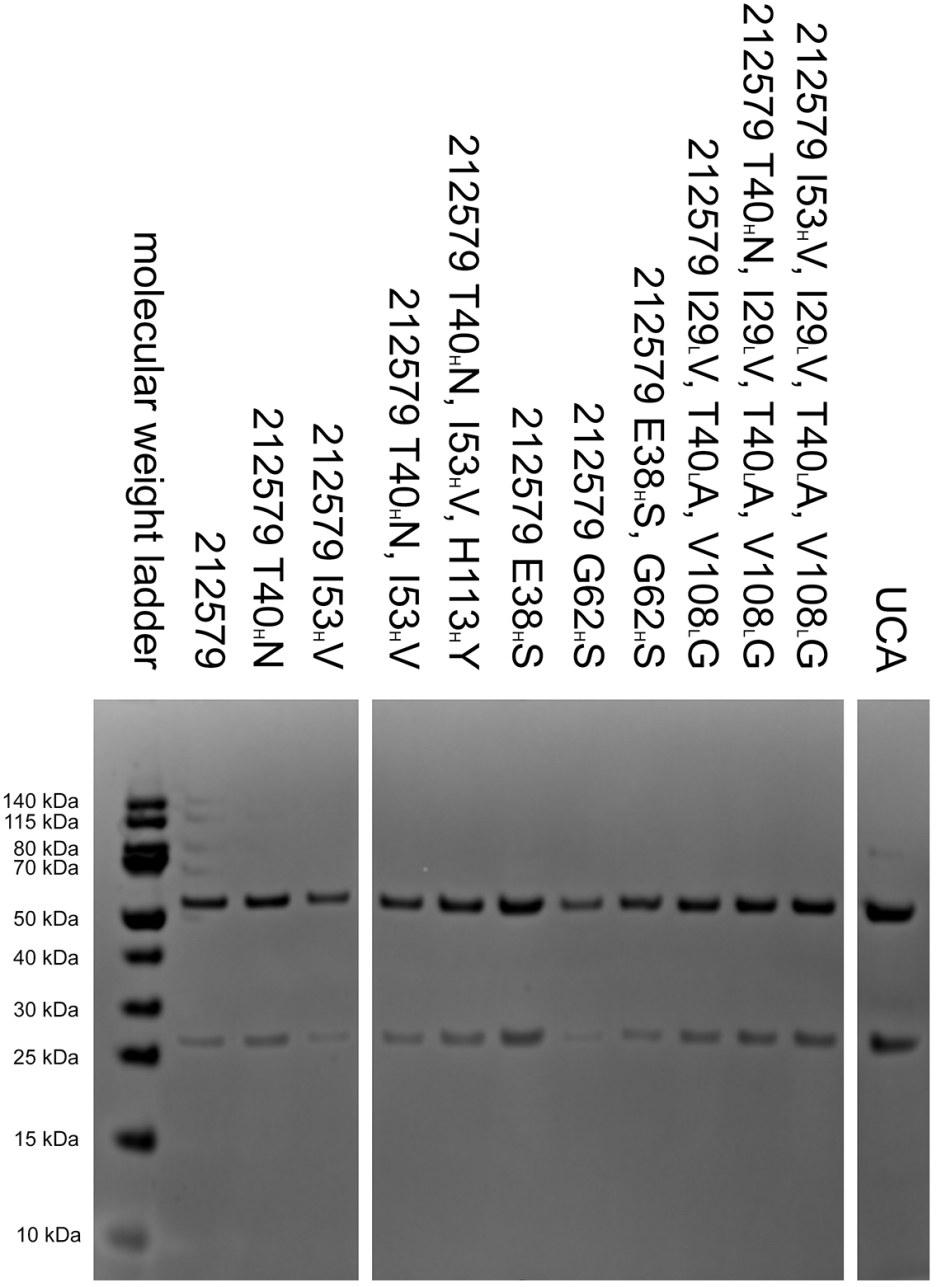
Reduced SDS-PAGE analysis of 212579, its inferred UCA and other variants of the clonotype after purification. Antibody 212579 H113_H_Y was also produced but achieved only low concentrations in culture supernatants. It was used for ELISA and affinity constant determination without prior purification.

**Figure 5:**
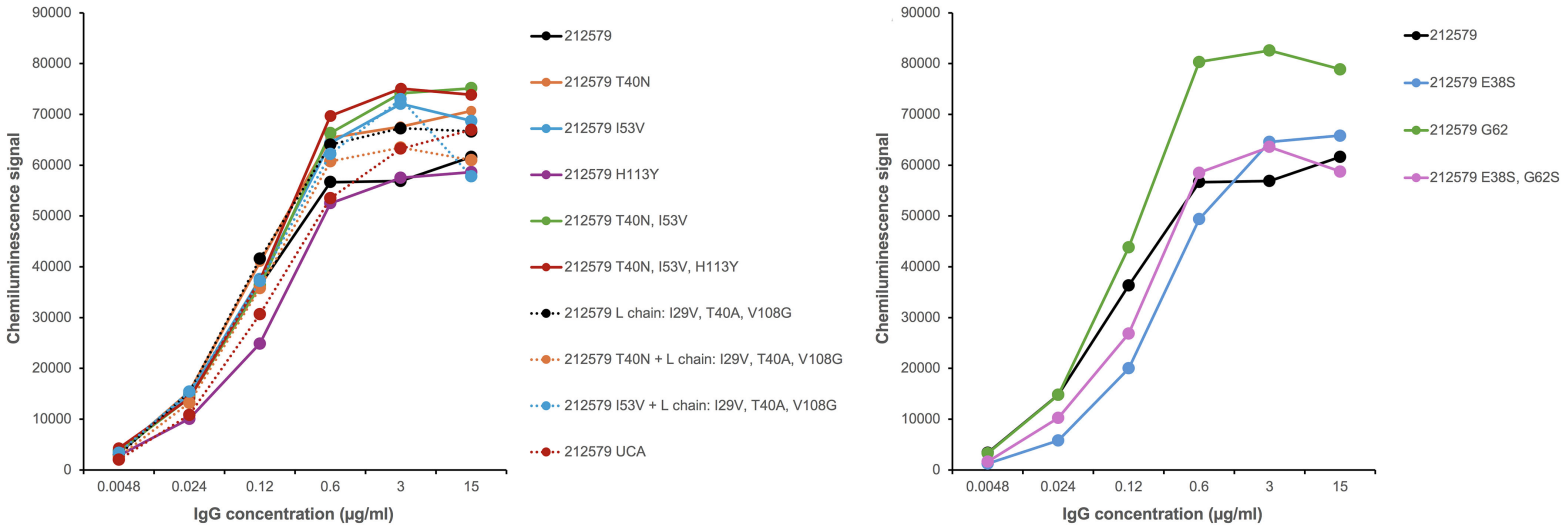
Antibody 212579 and recombinant variants thereof bind similarly to Phl p 5 immobilized onto a microtiter-plate surface in an assay format that allows for binding enhanced by avidity effects.

To assess the potential of an inferred UCA of antibody 212579 and various mutated intermediates to bind Phl p 5, we generated a set of reversion mutants of antibody 212579 in IgG1 format. All but one (212579 H113_H_Y) of the constructs produced well in Expi293F suspension cell culture in amounts sufficient for intact antibodies to be purified. Such antibodies were pure as determined by SDS-PAGE (**Figure 4**). All antibody variants bound Phl p 5 immobilized onto microtiter plates (**Figure 5**). Measurements of the affinity of the antibodies demonstrated that the antibody had affinity-matured about 27-fold from the inferred UCA to the 212579 antibody, but even the inferred UCA showed an affinity for the allergen in the low-nM range (**Table 2**). Germline gene allele IGHV3-48*03 and IGKV3-20*01 in combination are thus, given appropriate rearrangements, able to form high affinity binders that may populate the naïve antibody repertoire.

**Table 2:** Reaction rate constants and dissociate constants of 212579, its inferred UCA, and other sequence variants of the clonotype.

Mutant	k_a_ (1/Ms) ×10^5^	k_d_ (1/s) ×10^-4^	K_D_ (M) ×10^-9^
212579	4.4	1.4	0.32
UCA (212579 T40_H_N, I53_H_V, H113_H_Y, I29_L_V, T40_L_A, V108_L_G)	0.6	5.1	8.5
212579 T40_H_N	3.2	1.6	0.49
212579 I53_H_V	4.2	1.3	0.31
212579 H113_H_Y	2.6	3.6	1.4
212579 T40_H_N, I53_H_V	26	1.4	0.56
212579 T40_H_N, I53_H_V, H113_H_Y	1.6	1.4	0.92
212579 I29_L_V, T40_L_A, V108_L_G	2.3	3.5	1.5
212579 I53_H_V, I29_L_V, T40_L_A, V108_L_G	2.2	3.3	1.5
212579 T40_H_N, I29_L_V, T40_L_A, V108_L_G	1.2	5.0	4.3
212579 E38_H_S	2.8	27.3	9.9
212579 G62_H_S	4.2	1.1	0.27
212579 E38_H_S, G62_H_S	1.3	26.2	19.6

### 3.3 The N40_H_T substitution seen in antibody 212579 negatively impacts the antibody’s thermal stability

**Figure 6:**
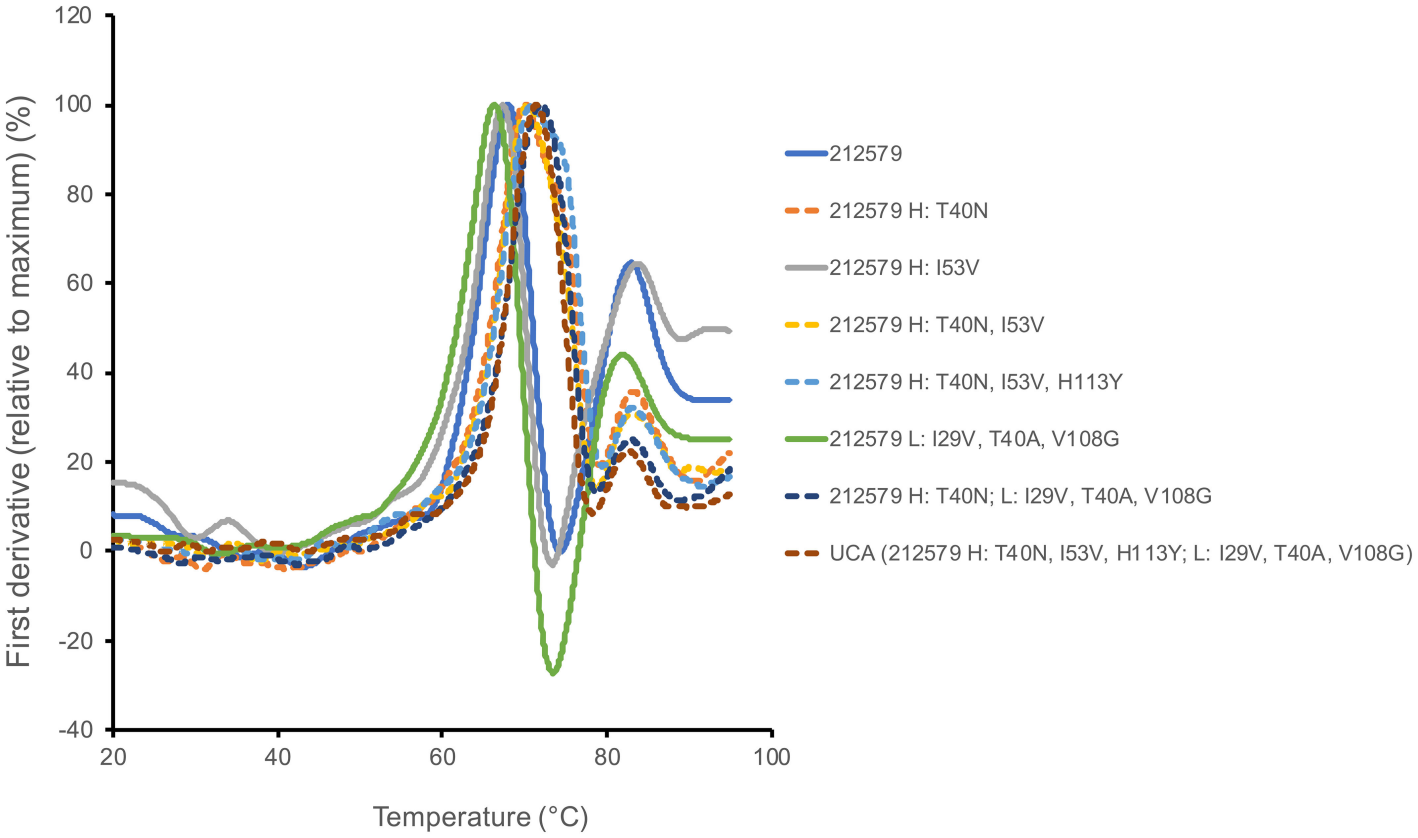
Differential scanning calorimetry illustrates differences in melting behavior between variants of antibody 212579 carrying T_H_40 (for instance as in antibody 212579; solid lines) or N_H_40 (for instance as in the inferred UCA of 212579; dashed lines). The curves represent the average of three independent runs.

**Figure 7:**
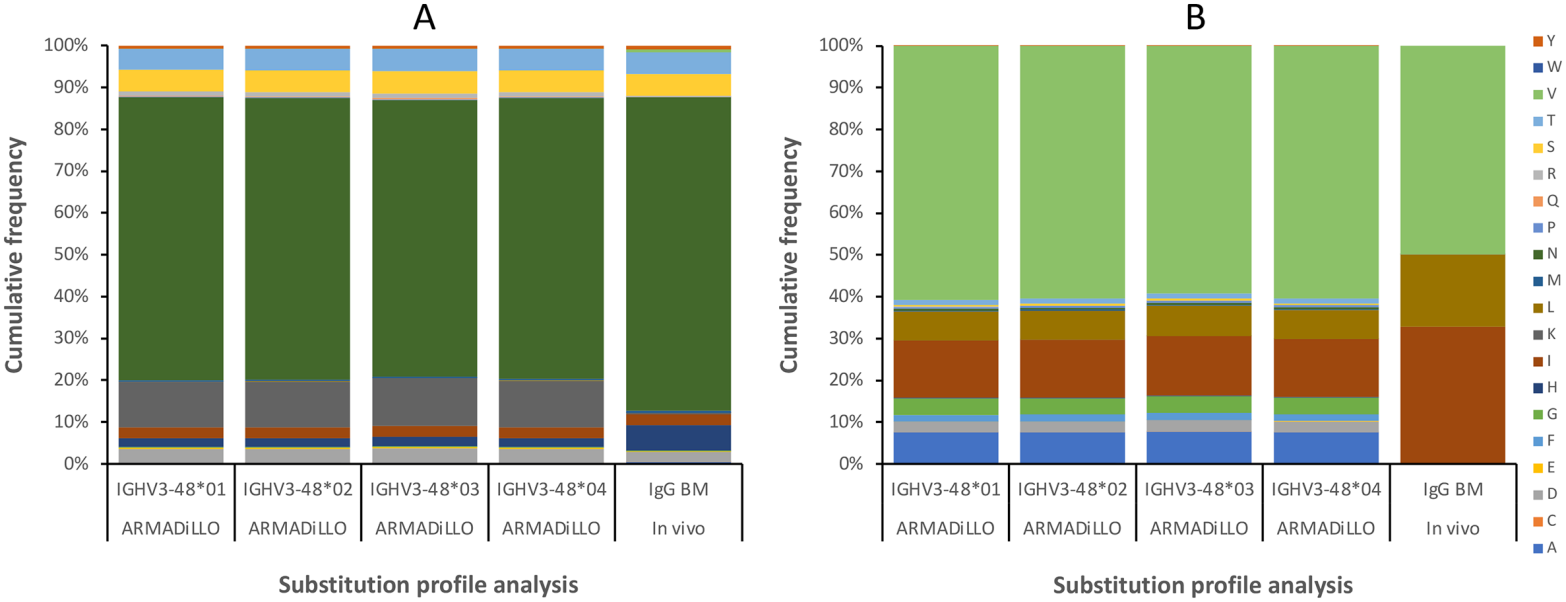
Substitution frequencies of residues 40_H_ **(A)** and 53_H_ **(B)**, as predicted by computational analysis (using the ARMADiLLO software tool (21, 22); 10% mutation level), and as observed in bone marrow (BM)-derived transcripts. Gene sequence diversity does not affect the computationally predicted substitution frequencies between alleles of IGHV3-48. *In vivo* data identifies the subset of the plausible substitutions that can be incorporated into positions 40_H_ and 53_H_ of antibodies derived from IGHV3-48, including substitutions N40_H_T and V53_H_I observed in 212579. Some substitutions are computationally predicted, based on the mutational patterns of the gene sequence (e.g. N40_H_K and multiple substitutions of residue V53_H_), but are not observed *in vivo*.

Antibody 212579 had a melting temperature Tm=67.8°C (**Table 3**, **Figure 6**). As outlined above it carries an N40_H_T substitution relative the inferred UCA. Residue 40 typically resides in the heavy/light chain domain interface. Computational assessment demonstrate the codon’s tendency to mutate so as to encode other amino acid residues (**Figure 7A**). Assessment of transcripts encoding IgG with an origin in IGHV3–48 suggests that substitutions of residue 40, including to T40_H_, are occasional occurrences in somatically hypermutated antibodies *in vivo* (**Figure 7A**). However, we demonstrate that this substitution negatively impacts thermal stability of the IgG as reversion of this mutation, alone or in combination with other reversions, increases the melting temperature of the antibody by 2.4-4.1°C (**Table 3**, **Figure 6**). In contrast, reversion of the other substitutions that occurred during the evolution of antibody 212579, including the commonly observed V53_H_I substitution (**Figure 7B**), has limited or no positive effect on the antibodies’ thermal stability.

**Table 3:** Melting temperature (Tm) and the hydrodynamic radius of 212579, its inferred UCA, and other sequence variants of the clonotype.

Mutant	Mean Tm ± SD (°C)	Hydrodynamic radius ± SD (nm)	
		25°C	37°C
212579	67.8 ± 0.0	6.52 ± 0.02	5.54 ± 0.15
UCA (212579 T40_H_N, I53_H_V, H113_H_Y, I29_L_V, T40_L_A, V108_L_G)	71.4 ± 0.0	6.28 ± 0.05	n.d.
212579 T40_H_N, I29_L_V, T40_L_A, V108_L_G	71.9 ± 0.2	6.11 ± 0.02	4.79 ± 0.03
212579 T40_H_N, I53_H_V, H113_H_Y	71.2 ± 0.2	6.32 ± 0.04	5.05 ± 0.04
212579 T40_H_N, I53_H_V	70.5 ± 0.1	6.03 ± 0.12	4.86 ± 0.13
212579 T40_H_N	70.2 ± 0.2	6.07 ± 0.02	4.89 ± 0.02
212579 I53_H_V, I29_L_V, T40_L_A, V108_L_G	68.3 ± 0.2	6.67 ± 0.02	5.24 ± 0.02
212579 I53_H_V	67.2 ± 0.1	6.20 ± 0.04	5.05 ± 0.08
212579 I29_L_V, T40_L_A, V108_L_G	66.2 ± 0.0	6.53 ± 0.02	5.05 ± 0.01
212579 E38_H_S	66.2 ± 0.0	6.49 ± 0.02	5.05 ± 0.02
212579 G62_H_S	65.7 ± 0.1	6.46 ± 0.06	5.20 ± 0.03
212579 E38_H_S, G62_H_S	68.3 ± 0.2	6.37 ± 0.02	4.92 ± 0.01

n.d., not determined

### 3.4 Residue 40_H_ impacts the hydrodynamic radius of the antibody

Antibody 212579, its inferred UCA, and their potential intermediates showed monodispercity with a hydrodynamic radius expected of IgG. Thus, none of them showed any tendency for aggregation. However, differences in their hydrodynamic radius were observed (**Table 3**). In particular, variants carrying residue N40_H_ had a hydrodynamic radius (6.16 ± 0.13 nm) at 25°C, lower than those of variants carrying T40_H_ (6.46 ± 0.15 nm) (p=0.015; Mann-Whitney test). Residue 40 in the heavy light chain interface thus impacts not only the thermal stability but also the overall structure of the antibody.

### 3.5 Structure model of antibody 212579 and its inferred UCA

**Figure 8:**
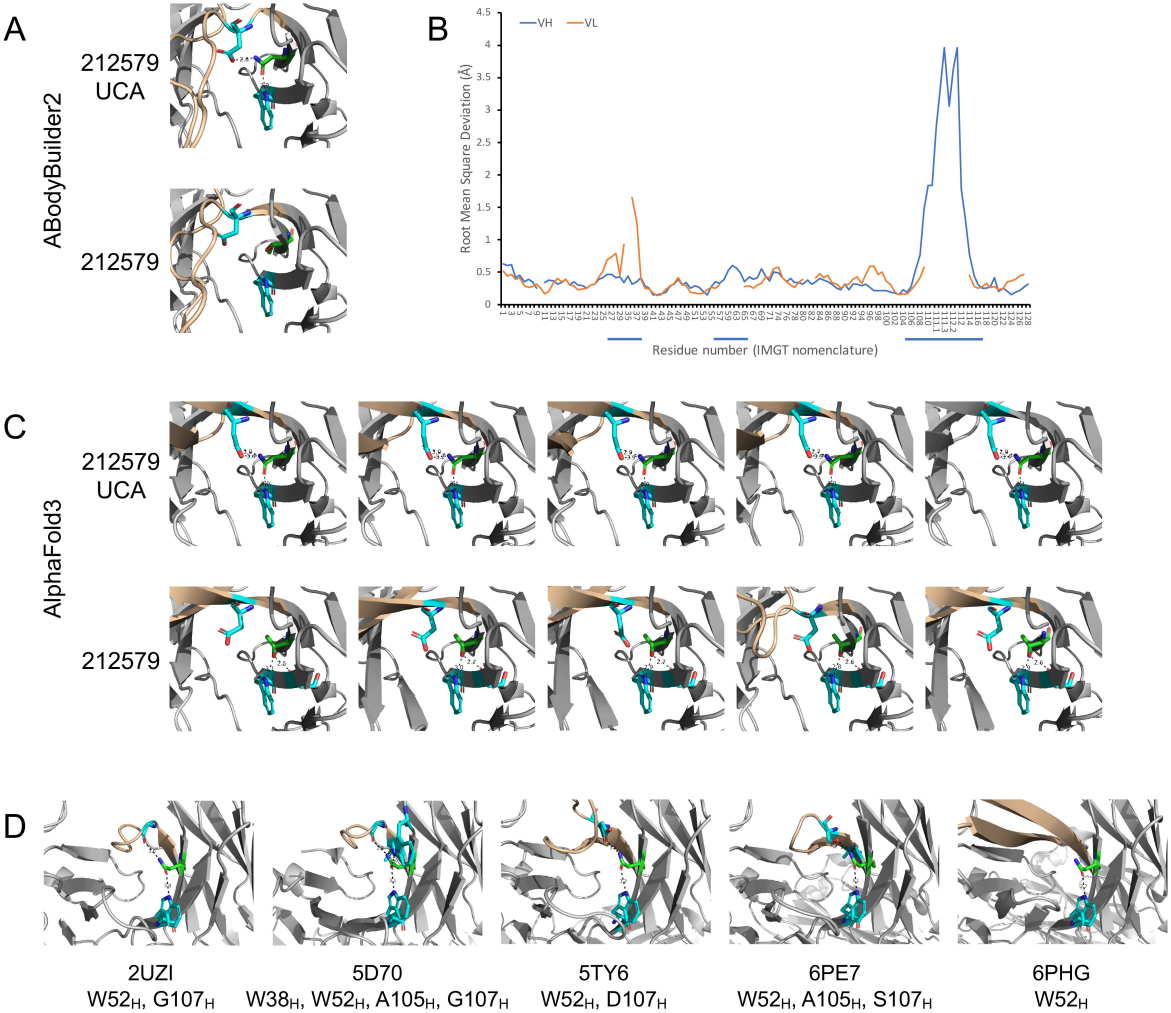
Model and crystal structures of antibodies derived from IGHV3-48. Antibody 212579 and its inferred UCA were modelled using ABodyBuilder2 **(A)** and AlphaFold3 **(C)**. The predicted error of each residue of the heavy and light chain variable domains (residues of CDRs are underlined) of the model of antibody 212579 as generated by ABodyBuilder2 is illustrated **(B)**. The backbone of CDR3 is highlighted in brown color. The modelled structures illustrate the position of residues W52_H_, D107_H_ (carbon: cyan) and residue 40_H_ (carbon: green) and proposed polar interaction made by the side chain of residue 40_H_. Other proposed polar interactions engaging the side chain of 40_H_ to the backbone oxygen of residue S54_H_ (carbon: cyan) are shown if implicated in the structures made by AlphaFold3. N40_H_ of the inferred UCA was in all cases proposed to make polar interactions with W52_H_ and D107_H_, while T40_H_ made no interaction with D107_H_ in any model proposed for 212579 by AlphaFold3 and no polar interactions with either W52_H_ or D107_H_ as proposed by ABodyBuilder2. **(D)** Crystal structures of antibodies (PDB: 2UZI, 5D70, 5TY6, 6PE7, and 6PHG) with a possible origin in IGHV3–48 and with residue N40_H_. The side chain of this residue makes polar interactions with W52_H_, and in 4/5 cases also with residues of the ascending strand of CDR3. Additional polar interactions of residue N40_H_ are also seen.

Structure models of antibody 212579 were generated using AlphaFold3 (14) and ABodyBuilder2 (15). While the overall backbone structure of the variable domains beyond heavy chain CDR3 agreed well between ABodyBuilder2 and AlphaFold3 (RMSDs of Cα: 0.28-0.35 Å), the proposed structure of the long CDR3 differed substantially between models proposed by the tools, in agreement with the challenges associated to computational prediction of the structure of antibody binding sites (25). For instance, the position of Cα of heavy chain CDR3 residue D111.3 of the consensus model proposed by ABodyBuilder2 is 14.9-21.1 Å away from its location in five models proposed by AlphaFold3. Substantial uncertainty of this hypervariable loop was also evident in the model generated by ABodyBuilder2 (**Figure 8B**) illustrating the challenge in defining the tip of this long loop by *in silico* modelling. The modelling tools were used to predict the effect of N40_H_T substitutions within the heavy-light chain interface of the heavy chain variable domain (**Figures 8A, C**). In line with the agreement of the backbone structure of much of the variable domains, both tools proposed the presence in the inferred UCA of a long-range network of polar interactions involving the side chains of N40_H_, W52_H_, and D107_H_, while the side chain of T40_H_ in antibody 212579 was proposed not to make polar interactions with D107_H_ (Alphafold3), or to engage in polar interactions at all (ABodyBuilder2) (**Figure 8A**). Importantly, studies of other human antibodies that carry N40_H_ also showed evidence of similar polar interaction networks in experimentally determined antibody structures engaging N40_H_, W52_H_, and residues 105_H_/107_H_ of the ascending strand of CDR3 (**Figure 8D**).

### 3.6 An allele-differentiating sequence variant at residue 38 is important for binding to the allergen

Immunoglobulin heavy chain variable germline gene IGHV3–48 is represented by four major alleles (**Figure 1**). These alleles are differentially represented in the population. For instance, in a Norwegian cohort (17), the IGHV3-48*03 allele is expressed in only 34/99 genotypes while alleles IGHV3-48*01 and IGHV3-48*02 dominate in the population. Unmutated antibodies encoded by IGHV3-48*03 differ from all other alleles of this gene by the presence of E38_H_ and G62_H_ instead of S38_H_ and S62_H_ (**Figure 1**). Introduction of both E38_H_S and G62_H_S substitutions, or one of these substitutions into antibody 212579 did not substantially affect protein production, product monodispersity, or the ability of the product to bind Phl p 5 in an immunoassay format that allows for bivalent binding to the immobilized antigen. Some reduction in melting temperature was seen, in particular for the G62_H_S variant but not for the E38_H_S G62_H_S substituted antibody. However, the E38_H_S (but not the G38_H_S) substitution substantially affected the monovalent affinity for the antigen (**Table 2**) primarily by increasing the dissociation rate of the complex approximately 20-fold. The presence of E38_H_ unique to allele IGHV3-48*03 is thus critical for the high-affinity antigen-binding property of the clonotype.

## 4 Discussion

Affinity is an important aspect of bioactivity of allergen-specific antibodies (7, 8). Antibody 212579, a Phl p 5 allergen-specific monoclonal antibody, has evolved *in vivo* through a minimal set of mutations into a high affinity, allergen-specific antibody. However, also its inferred UCA was shown to display a high affinity for the antigen in the low-nM range. The present study thus illustrates how a high affinity binder to a major allergen is readily achieved even without mutation in some clonal lineages and further evolved into sub-nM affinity through a minimal number of substitutions. As only a fraction of IgE antibodies needs to be of high affinity to induce efficient basophil degranulation (2, 7), these findings illustrate how such a population of specific binders can be readily achieved without the need for extensive affinity maturation. Interestingly, as antibody 212579, an antibody of the IgG1 subclass, is a member of a clonal lineage that also harbors more mutated IgE antibodies of similar affinity (9), it also illustrates the potential of clonal development of high affinity antibodies with potential to block basophil activation (7, 8).

All investigated variants of 212579 bound the allergen well in an assay format that allows for avidity effects based on binding of a divalent antibody to antigen immobilized onto a surface. Assessment of monovalent binding interaction, while demonstrating the low-nM affinity even of the inferred UCA of antibody 212579, illustrated that antibody 212579 had enhanced its affinity toward the allergen, by improvement of both the association and dissociation rate constants. None of the substitutions of antibody 212579 were on their own responsible for the affinity enhancement. Reversion of the three substitutions of the light chain variable domain, that only target one residue commonly being a contact residue (26), reduced the affinity 4.7-fold. While reversion of the substitution of heavy chain variable domain residues 40 and 53 did not substantially affect binding affinity, reversion of the substitution of residue 113 reduced affinity 4.3-fold. Among the residues of the heavy chain that were affected by substitutions, only residue 113 is a common antigen contact residue (26), while residues 40_H_ and 53_H_ reside in the heavy/light chain variable domain interface and the lower domain core, respectively. These substitutions, N40_H_T and V53_H_I, are computationally predicted to occur during somatic hypermutation and are occasionally and commonly, respectively, seen in IgG repertoires (**Figure 6**). Past studies proposed that substitution of residue N40_H_ might, through its ability to make polar interaction with D107H, have impact on the paratope (24). Here we provide experimental evidence that, although no effect on the affinity to the antigen was observed upon mutation of residue 40, such substitutions influenced the thermal stability of the protein and its hydrodynamic radius. Residue 40, residing in the heavy-light chain interface, thus represents a viable candidate for optimization of antibodies, including those developed for use in allergy immunotherapy, for instance in cases where developability issues are associated to the protein product.

The present study illustrates that alleles IGHV3-48*03 and IGKV3-20*01 are capable of generating a high affinity (low-nM) binder also in the naïve, unmutated stage. IGKV3-20*01 is highly expressed and present at high frequency in the human population while IGHV3-48*03 is present only in about 1/3 of subjects in this Scandinavian population. Residue E38_H_, a residue that commonly is engaged in antibody-protein antigen interactions (26), unique to this allele of IGHV3-48, is highly critical for the high affinity nature of antibody 212579 as substitution of this residue alone to serine, as found in other alleles of IGHV3-48, reduced the affinity for the allergen approximately 20-fold. We hypothesize that subjects carrying the IGHV3-48*03 allele in their genotype, might be poised to develop antibodies to the epitope of Phl p 5 recognized by antibody 212579, in particular as alleles of IGHD, IGHJ, IGKV, and IGKJ engaged in creation of the inferred 212579 UCA (**Figure 2**) are frequently present in human genotypes.

Interestingly, antibody responses, if deconvoluted to epitope specificity, are commonly generated by a limited set of immunoglobulin rearrangements. The responses to the CD4-binding site of human immunodeficiency virus-1, the stem of influenzae virus hemagglutinin, and the AD-2 epitope of cytomegalovirus glycoprotein B, responses that are enriched in sequences derived from germline genes IGHV1-2 (27–29), IGHV1-69 (30), and IGHV3–30 and closely related genes thereof (31, 32), respectively, and in some cases even particular alleles thereof, are other prime examples of such stereotyped responses. Also antibody responses to certain allergens have been shown to be germline gene-restricted including responses to peanut allergen Ara h 2 (10, 11), and grass pollen allergen Phl p 2 (33–35). The immune response to Phl p 5 is, however, known to target multiple epitopes (36) and to be highly diverse in genetic origin (37–39). Given the limited information on gene usage of antibodies targeting individual epitopes it is still unknown if particular epitopes of Phl p 5 are primarily recognized by antibodies of a limited set of germline gene or allele origins. Grass pollen allergen epitope recognition profiles are, however, highly individualized (40, 41). It thus remains to be investigated if such personal epitope recognition patterns are associated to each subjects’ immunoglobulin germline gene/allele repertoire, for instance as exemplified by allelic differences of IGHV3-48.

In summary, we have identified an inferred UCA of the allergen-specific antibody 212579 that displays high affinity for its target allergen. It illustrates the potential of unmutated naïve antibodies of the immune repertoire to recognize the major allergen Phl p 5, a route that offers a shortcut to the development of antibodies with potential to induce an allergic condition or (as IgG) to block basophil activation. Residue N40_H_ of the germline gene was identified as a residue that, potentially through a hydrogen-bonded network, stabilize the antibody structure. As this residue is occasionally mutated in human antibodies, we postulate that reversion of such substitutions may be a viable approach to enhance for instance the developability character of antibodies. Finally, we demonstrate that residue E38_H_, a unique residue of allele IGHV3-48*03, substantially contributed to the high affinity of the clonotype, suggesting an allele-differentiating effect in targeting the epitope of this major allergen.

## Data Availability

The sequences of the 212579 antibody and its inferred UCA presented in this study can be found in online repositories. The names of the repository/repositories and accession number(s) can be found in the article.
